# Consumed Foodstuffs Have a Crucial Impact on the Toxic Activity of Enteropathogenic *Bacillus cereus*

**DOI:** 10.3389/fmicb.2018.01946

**Published:** 2018-08-17

**Authors:** Claudia Da Riol, Richard Dietrich, Erwin Märtlbauer, Nadja Jessberger

**Affiliations:** Department of Veterinary Sciences, Faculty of Veterinary Medicine, Ludwig-Maximilians-Universität München, Munich, Germany

**Keywords:** *Bacillus cereus*, food infections, stomach passage, survival, cytotoxicity

## Abstract

Enteropathogenic *Bacillus cereus* cause diarrhea due to the production of enterotoxins in the intestine. To start this process, spores have to be ingested together with contaminated food and survive the stomach passage. In this study, the influence of consumed foodstuffs on spore survival as well as on cytotoxicity toward colon epithelial cells was investigated. Spore survival of 20 enteropathogenic and apathogenic *B. cereus* strains during simulated stomach passage was highly strain-specific and did not correlate with the toxic potential. Survival of three tested strains was strain-specifically altered by milk products. Whereas milk, a follow-on formula and rice pudding had only little influence, spores seemed to be protected by milk products with high fat content such as whipped cream and mascarpone. Furthermore, tested milk products decreased the toxic activity of three *B. cereus* strains toward CaCo-2 cells. Investigating the individual components, lactoferrin, a skim milk powder and vitamins C, B5 and A showed the most inhibiting effects. On the other hand, biotin, vitamin B3 and another skim milk powder even enhanced cytotoxicity. Further studies suggested that these inhibiting effects result only partially from inhibiting cell binding, but rather from blocking the interaction between the single enterotoxin components.

## Introduction

Foodborne infections become increasingly attributed to *Bacillus cereus* (Mead et al., 1999; Scallan et al., 2011; Anonymous, 2013). The Gram-positive, ubiquitous spore-former causes two types of gastrointestinal diseases, the emetic and the diarrheal syndrome (Stenfors Arnesen et al., 2008; Bottone, 2010). Emetic strains produce the heat-stable cyclic dodecadepsipeptide cereulide leading to nausea and vomiting (Agata et al., 1995; Ehling-Schulz et al., 2004; Messelhäusser et al., 2014). The diarrheal form is caused by heat-labile enterotoxins, which are the single protein CytK (cytotoxin K; Lund et al., 2000) and the two three-component complexes Nhe (non-hemolytic enterotoxin; Lund and Granum, 1996; Didier et al., 2016) and Hbl (hemolysin BL; Beecher et al., 1995). In routine diagnostics, the enteropathogenic potential of a new isolate is so far determined via its ability to produce enterotoxins after growth under standard laboratory conditions (Jessberger et al., 2015). However, in the meantime *B. cereus* food infections are rather understood as multifactorial processes including various steps such as (i) the survival of spores during stomach passage (Clavel et al., 2004; Wijnands et al., 2009; Ceuppens et al., 2012a; Berthold-Pluta et al., 2015), (ii) germination of spores in the small intestine (Clavel et al., 2004; Wijnands et al., 2006, 2007),(iii) motility (Ghelardi et al., 2007; Salvetti et al., 2007; Mazzantini et al., 2016), (iv) adhesion of spores and vegetative cells to the intestinal epithelium (Minnaard et al., 2004; Ramarao and Lereclus, 2006), and afterward (v) the production of enterotoxins under intestinal conditions (Jessberger et al., 2017). Furthermore, the impact of complex foodstuffs has also been tested, mainly focusing on bacterial survival. Clavel and coworkers investigated survival of *B. cereus* spores and vegetative cells in artificial stomach fluid after addition of half-skim milk, pea soup or chicken (Clavel et al., 2004). In another study cauliflower, broccoli and okara byproducts limited growth of different foodborne bacteria, amongst them *B. cereus* (Sanz-Puig et al., 2015). Very little is known about the effect of ingested foodstuffs on the toxic activity of *B. cereus* toward colon epithelial cells. In a first study it was shown that kefir antagonizes the cytopathic effects of *B. cereus* toward CaCo-2 cells without influencing adhesion (Medrano et al., 2008, 2009).

Here we report that consumed milk products or their single components have important impact not only on the survival of *B. cereus* spores during stomach passage, but also on the toxic activity of the *B. cereus* enterotoxins toward colon epithelial cells.

## Materials and Methods

### Bacterial Strains and Culture Conditions

In this study, a set of 20 enteropathogenic and apathogenic *B. cereus* strains was used, which has been extensively characterized (Kniehl et al., 2003; Jessberger et al., 2015, 2017). For toxicity studies, a *nheABC* deletion strain (Tausch et al., 2017) was additionally used. Overnight cultures were grown in CGY medium (casein-glucose-yeast: 2% casein hydrolysate, 0.6% yeast extract, 0.2% ammonium sulfate, 1.4% K_2_HPO4, 0.6% KH_2_PO4, 0.1% sodium citrate × 2H_2_O, 0.2% MgSO_4_ × 7H2O, 1% glucose) at 32°C under continuous agitation. For collection of toxin-rich supernatants, cells were treated as previously described (Jessberger et al., 2014).

### Cell Lines and Culture Conditions

CaCo-2 cells were obtained from DSMZ (German Collection of Microorganisms and Cell Cultures, Braunschweig, Germany), cultivated in 80 cm^2^ culture flasks in a humidified incubator at 37°C and 7% CO_2_ and passaged 1:6 every 3–4 days. RPMI 1640 medium (with stable glutamine; Biochrom AG) was supplemented with 10% fetal bovine serum (Biochrom AG).

### Spore Preparation

2 × 100 ml fresh Minimal Sporulation Medium (1 M MgCl_2_ × 6 H_2_O, 1 M Ca(NO_3_)_2_ × 4 H_2_O, 12.5 mM ZnCl_2_, 2.5 mM CuCl_2_, 2.5 mM CoCl_2_ × 6 H_2_O, 2.5 mM Na_2_MoO_4_ × 2 H_2_O, 5 mM (NH_4_)_2_SO_4_, 0.066 mM MnSO_4_ × H_2_O, 1 mM FeSO_4_ and 1 M maltose in bacto nutrient broth (Oxoid); pH 7.6) were inoculated with 0.5% overnight culture of each *B. cereus* strain. After 3 days incubation at 30°C under continuous shaking, >80% spores were present. The cultures were centrifuged (4,000 g, 7 min, 4°C) and washed three times in pre-cooled Spore Washing Buffer (2 mM KH_2_PO_4_, 8 mM K_2_HPO_4_, pH 7.4). Spore suspensions were stored at 4°C and further washed once a week for 3 weeks. Immediately before an experiment, spores were washed once again. To determine spore concentrations, suspensions were incubated for 10 min at 80°C to eliminate residual vegetative cells. Serial dilutions of untreated and heat-treated spores were plated on CGY agar plates. After overnight incubation at 32°C, cfu was determined.

### Survival of the Stomach Passage

Survival of *B. cereus* spores in a medium mimicking the human stomach was investigated following (Clavel et al., 2004). Fifty milliliter pre-warmed medium (4.8 g/l NaCl, 1.56 g/L NaHCO_3_, 2.2 g/l KCl, 0.22 g/l CaCl_2_ and 500 U/l Pepsin; pH 2) were inoculated with 10^7^ *B. cereus* spores. If appropriate, different foodstuffs were added in 1:1 ratio to the medium (see below). The mixture was incubated for 6 h at 37°C under smooth shaking. pH 2 was maintained during the entire experiment. Every hour 300 μl samples were taken and incubated at 80°C for 10 min. Subsequently, serial dilutions were plated on CGY agar. After overnight incubation at 32°C, cfu was determined and compared with the inoculum, which was also plated on CGY agar.

### Food Preparation

To assess the impact of foodstuffs on spore survival, different milk products were tested: UHT milk 1.5%, UHT milk 3.5%, UHT milk 1.5% lactose-free, UHT whipped cream (30% fat content), mascarpone (composition: pasteurized cream, pasteurized milk, acidifier: citric acid, rennet, lactic acid bacteria; 80% fat in dry mass), a baby follow-on formula (composition: see **Table 1**) and rice pudding (composition: milk 3%, H_2_O, rice, sugar, modified starch, salt, aroma, no additional fruits). All products were pre-tested for total bacterial counts (according to DIN 10192) and presumptive *B. cereus* (according to DIN 10198). Only contamination-free products were used for the experiments. The baby follow-on formula (14.1 g) was dissolved in 90 ml a. dest. according to the instructions of the manufacturer. The rice pudding was homogenized for 2 min in a stomacher homogenizer. Finally, 50 g or ml food were mixed in a 1:1 ratio with the stomach medium (Clavel et al., 2004). After that, pH values were adjusted to 2 by using 5 M HCl.

**Table 1:** Foodstuffs and food components used in WST-1 bioassays in this study.

	Concentration in WST	Influence on toxicity	Effect in EIA NheA NheB Hbl L2 Hbl L1 Hbl B				
**Milk products**							
UHT milk 1.5%	2%	**↓**	+	**−**	**−**	+	+
UHT milk 3.5%	2%	**↓**	+	**−**	**−**	+	+
UHT lactose-free milk 1.5%	2%	**↓**	+	**−**	**−**	+	**−**
Baby follow-on formula	3.925 mg/ml	**↓**	+	**−**	**−**	+	+
**Milk components**							
α-casein	0.2%	**↓**	**−**	**−**	**−**	**−**	+
β-casein	0.2%	**−**	n.d.	n.d.	n.d.	n.d.	n.d.
κ-casein	0.2%	**−**	n.d.	n.d.	n.d.	n.d.	n.d.
α-lactalbumin	0.2%	**↓**	**−**	**−**	**−**	**−**	**−**
β-lactoglobulin	0.2%	**−**	n.d.	n.d.	n.d.	n.d.	n.d.
Lactoferrin	0.2%	**↓**	**−**	**−**	**−**	**−**	**−**
**Follow-on formula components**							
Skim milk powder I	5 mg/ml	**↓**	**−**	**−**	**−**	+	+
Skim milk powder II	5 mg/ml	**−**	n.d.	n.d.	n.d.	n.d.	n.d.
Skim milk powder III	5 mg/ml	**↑**	**−**	**−**	**−**	+	**−**
Starch	9.06 μg/ml	**−**	n.d.	n.d.	n.d.	n.d.	n.d.
Calcium carbonate	5 μg/ml	**−**	n.d.	n.d.	n.d.	n.d.	n.d.
Vitamin C (L-ascorbic acid)	364 μg/ml	**↓**	**−**	**−**	**−**	**−**	**−**
Potassium chloride	1.9 mg/ml	**−**	n.d.	n.d.	n.d.	n.d.	n.d.
Ferric lactate	9.77 μg/ml	**−**	n.d.	n.d.	n.d.	n.d.	n.d.
Vitamin E^∗^	1.19 mg/ml	**−**	n.d.	n.d.	n.d.	n.d.	n.d.
Zinc sulfate	50.88 μg/ml	**−**	n.d.	n.d.	n.d.	n.d.	n.d.
Vitamin B5 (calcium-D-panthotenate)	1.24 mg/ml	**↓**	**−**	**−**	**−**	**−**	**−**
Copper sulfate	2 μg/ml	**−**	n.d.	n.d.	n.d.	n.d.	n.d.
Vitamin A (retinol)^∗^	3.91 μg/ml	**↓**	+	**−**	**−**	**−**	**−**
Vitamin B1 (thiamine)	316 μg/ml	**−**	n.d.	n.d.	n.d.	n.d.	n.d.
Vitamin B6 (pyridoxine hydrochloride)	1.319 mg/ml	**−**	n.d.	n.d.	n.d.	n.d.	n.d.
Manganese sulfate	0.625 μg/ml	**−**	n.d.	n.d.	n.d.	n.d.	n.d.
Potassium iodate	788 μg/ml	**−**	n.d.	n.d.	n.d.	n.d.	n.d.
Folic acid	125 μg/ml	**−**	n.d.	n.d.	n.d.	n.d.	n.d.
Vitamin K1 (phytomenadione)^∗^	615 μg/ml	**−**	n.d.	n.d.	n.d.	n.d.	n.d.
Sodium selenate	4.14 μg/ml	**−**	n.d.	n.d.	n.d.	n.d.	n.d.
Biotin	5 μg/ml	**↑**	−	−	−	−	−
Vitamin D3^∗^	3.91 μg/ml	−	n.d.	n.d.	n.d.	n.d.	n.d.
Vitamin B3 (nicotinic acid)	313 μg/ml	**↑**	−	−	−	−	−

*In general, 2% milk was added to the cell culture medium, milk components were adjusted to 0.2%. Follow-on formula components were initially added in the highest concentration which was solvable, did not affect the CaCo-2 cells and did not interfere with the photometric measurement. ^∗^ Stock solution in 70% ethanol. ↓: decrease of toxic activity. ↑ increase of toxic activity. + Altered toxin titers in EIA. - Low or no influence on toxicity and EIA results. n.d., not determined.*

### WST-1 Bioassay

WST-1 bioassays were performed on CaCo-2 cells as described earlier (Jessberger et al., 2017). Briefly, serial dilutions of the culture supernatants were applied to 96 well plates in RPMI 1640 medium (100 μl/well). Food components were added in constant concentration to the medium as indicated in **Table 1**. Subsequently, 2 × 10^4^ 3 day old CaCo-2 cells/well (100 μl) were applied. After 24 h incubation at 37°C and 7% CO_2_, cell viability was determined by addition of WST-1 (Roche diagnostics). OD_450nm_ was recorded in a Tecan infinite F50 photometer. Dose-response curves and thus 50% lethal concentrations were calculated with Magellan software and are shown as reciprocal titers.

### Propidium Iodide (PI) Influx Tests

To assess pore formation in the membranes of CaCo-2 cells, propidium iodide influx studies were performed as previously described (Jessberger et al., 2014, 2017). Briefly, 4 × 10^4^ 3 day old CaCo-2 cells were grown overnight in 96 well plates. For consecutive application of foods and *B. cereus* supernatants, RPMI 1640 medium was removed and 200 μl fresh medium containing food components in concentrations as stated in **Table 1** were added for 1 h. After three washing steps in RPMI 1640 medium, 200 μl fresh medium were added containing 10 μg/ml PI (Sigma-Aldrich) and 1:40 dilutions of the culture supernatants. For consecutive application of recombinant Hbl proteins (Tausch et al., 2017), stock solutions (1.5 pmol/μL) of each component were mixed 1:40 in RPMI 1640 medium (alone or with food components in concentrations as stated in **Table 1**). After application of Hbl B and L1, CaCo-2 cells were washed three times in RPMI 1640 medium. Hbl L2 was applied in medium additionally containing 10 μg/ml PI. Immediately after addition of PI, fluorescence was measured in a Victor 1420 multilabel counter (Perkin Elmer) for 4 h every 2.5 min (excitation: 530 nm; emission: 616 nm; excitation time: 1 s; excitation strength: 20,000).

### Enzyme Immunoassays (EIAs)

Sandwich enzyme immunoassays for the detection of enterotoxin components were carried out as previously described (Dietrich et al., 1999, 2005; Jessberger et al., 2015, 2017; Tausch et al., 2017). Food components were dissolved in PBS in concentrations as stated in **Table 1** and mixed 1:1 with *B. cereus* culture supernatants. The following antibodies were used for detection: 5 μg/ml mAb 2B11/1E11-HRP 1:4,000 (NheB), 2.5 μg/ml mAb 1G4/2G11-HRP 1:2,000 (NheA), 10 μg/ml mAb 1A12/1H9-HRP 1:2,000 (Hbl L2), 5 μg/ml mAb 1E9/1G8-HRP 1:2,000 (Hbl L1), and 2.5 μg/ml mAb 1D12/1B8-HRP 1:2,000 (Hbl B). Titers are defined as the reciprocal of the highest dilutions resulting in an absorbance value of ≥1.0. Results were compared according to those titers and are summarized in **Table 1**.

## Results

### Survival of the Stomach Passage Is Highly Strain-Specific

**FIGURE 1:**
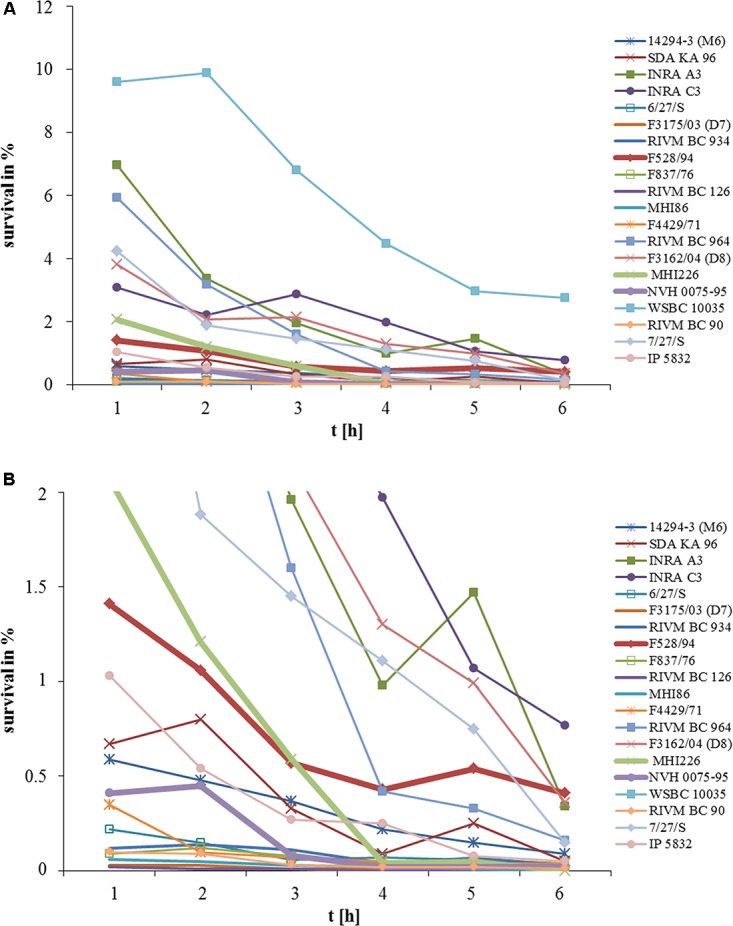
Survival of 20 *B. cereus* strains in medium simulating the stomach passage. Medium (Clavel et al., 2004) was inoculated with 10^7^ spores. Every hour, samples were taken and cfu was determined. **(A)** Survival is shown as % of the inoculum. **(B)** Enlargement of data points obtained for survival rates between 0 and 2%.

In a preliminary experiment, 20 *B. cereus* strains were tested for their ability to survive conditions mimicking the stomach (Clavel et al., 2004). The strain set consists of enteropathogenic (highly toxic) and apathogenic or low toxic isolates, which have previously been characterized in detail (Kniehl et al., 2003; Jessberger et al., 2015, 2017). Spore preparations were used for inoculation, as vegetative cells did not survive under the chosen conditions (data not shown). Spores of all 20 strains were able to survive for at least 1 h (**Figure 1**). Highest rates were found for WSBC 10035 (highly toxic; 9.6%) as well as for INRA A3 (low toxic), RIVM BC 964 (highly toxic) and 7/27/S (highly toxic) (all >4%). RIVM BC 126 (highly toxic) showed poorest survival (0.02%). After 6 h, survival of 19 strains dropped to <1% (**Figure 1**). Thus, the ability of *B. cereus* spores to resist the stomach conditions depends massively on the individual strain. Furthermore, survival does not correlate with the toxic potential of an isolate.

### Protection of *B. cereus* Spores by Foodstuffs

**FIGURE 2:**
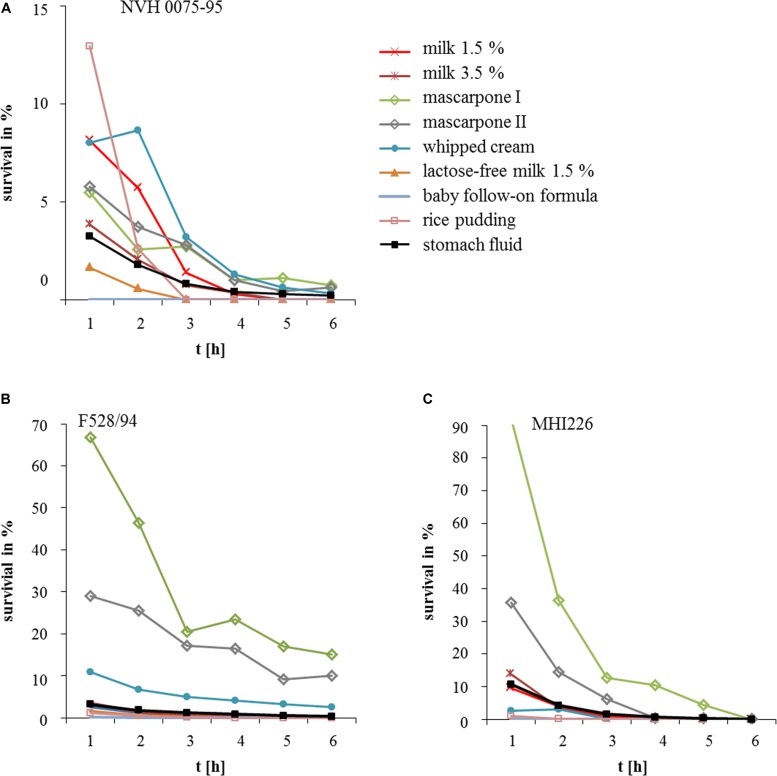
Survival of *B. cereus* in stomach medium with different foodstuffs. **(A)** *B. cereus* strain NVH 0075-95 (highly toxic, highly susceptible toward the stomach medium, see **Figure 1** and Jessberger et al., 2015, 2017). **(B)** *S*train F528/94 (low toxic, medium susceptibility toward the stomach medium, see **Figure 1** and Jessberger et al., 2015, 2017). **(C)** Strain MHI 226 (low toxic, medium susceptibility toward the stomach medium, see **Figure 1** and Jessberger et al., 2015, 2017). Strains were grown for 6 h in stomach fluid (Clavel et al., 2004) as control and additionally with milk (1.5 and 3.5% fat), 1.5% lactose-free milk, whipped cream, mascarpone, baby follow-on formula and rice pudding. Survival rates are comparatively shown. Survival is depicted as% of the inoculum.

To investigate the impact of ingested foodstuffs on spore survival, the highly toxic reference strain NVH 0075-95 (*nhe*+, low survival rate) was chosen, as well as the two low toxic strains F528/94 (*nhe*+, *hbl*+) and MHI 226 (*nhe*+) (Jessberger et al., 2015), both with medium survival rates as shown in **Figure 1**. As we were especially interested in milk and milk products, we tested milk 1.5%, milk 3.5%, lactose-free milk 1.5%, whipped cream, mascarpone, a baby follow-on formula and rice pudding. As above, spore survival was tested for 6 h. The effect of the foodstuffs on spore survival was highly variable depending on the individual *B. cereus* strain (**Figure 2**). While reference strain NVH 0075-95 showed comparably low survival rates with all foodstuffs tested (**Figure 2A**), spores of strain F528/94 were protected by whipped cream and especially mascarpone (**Figure 2B**). Mascarpone also significantly increased survival of spores of strain MHI 226, while lactose-free milk, the follow-on formula and rice pudding even seemed to impair spore survival (**Figure 2C**). Two mascarpone samples were investigated. Mascarpone I, which protected the spores even more clearly, was at the moment the experiment was conducted already expired by 2 weeks, while mascarpone II was within the best before date. Generally it can be postulated that, though highly strain-specific, *B. cereus* spores survive the stomach environment more easily in the presence of more complex milk products with higher fat content.

### Food Components Influence the Toxic Activity of *B. cereus* Supernatants

**FIGURE 3:**
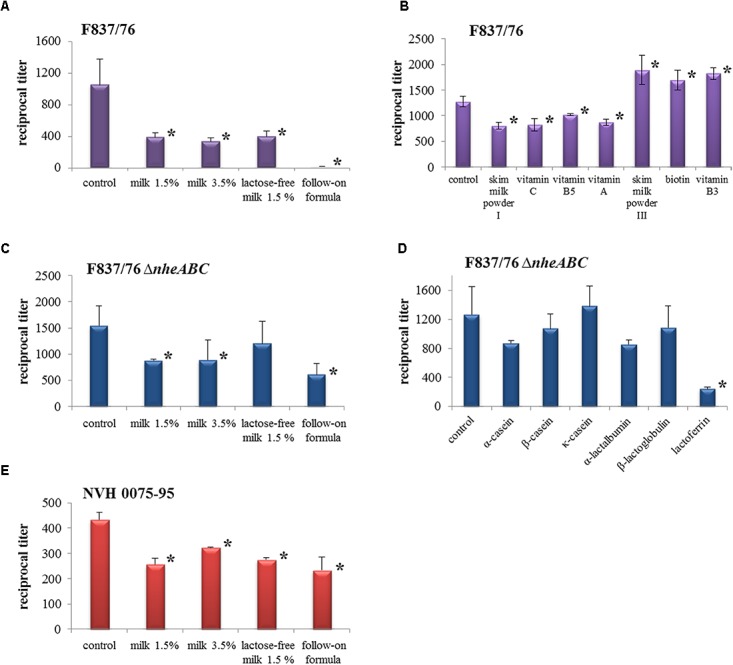
Cytotoxic activity of *B. cereus* culture supernatants in the presence of different milk products. Results of WST-1 bioassays performed on CaCo-2 cells are shown as reciprocal titers. In these experiments, *B. cereus* supernatants were applied as serial dilutions, while foodstuffs were added in constant concentrations as stated in **Table 1**. **(A)** Reference strain F837/76 (*nhe*+, *hbl*+) with foods. **(B)** Cytotoxicity of strain F837/76 under addition of different follow-on formula ingredients. The positive control differs from that in **A**, as it was obtained in an independent approach. **(C)** *nheABC* deletion mutant of F837/76 (*hbl*+). **(D)** Cytotoxicity of the *nheABC* deletion strain under addition of milk proteins. The positive control differs from that in **(C)**, as it was obtained in an independent approach. **(E)** Reference strain NVH 0075-95 (*nhe*+). ^∗^Significant difference (*P*-value < 0.05). Data were statistically analyzed using the column statistics program (unpaired *t*-test, 95% confidence interval) of Graph Pad Prism version 5.00 (GraphPad Software, La Jolla, CA, United States).

To show that foodstuffs cannot only influence spore survival, but also the activity of the *B. cereus* enterotoxins, WST-1 bioassays were performed on CaCo-2 cells. Here, mascarpone, whipped cream and rice pudding could not be further investigated due to the high susceptibility of the cells and the test system. The remaining foodstuffs were added to the test in concentrations as stated in **Table 1**. **Figure 3** shows that the toxic activity of three *B. cereus* strains was significantly decreased by milk and the baby follow-on formula. Toxicity titers of reference strain F837/76, which secretes both enterotoxins, Nhe and Hbl, were halved in the presence of milk 1.5%, milk 3.5% and lactose-free milk and even reduced to a minimum in the presence of the baby follow-on formula (**Figure 3A**). Toxicity of strain F837/76 Δ*nheABC*, which is able to produce only Hbl, was also reduced. Again, the baby follow-on formula had the strongest impact (reduction approximately 66%), whereas lactose-free milk reduced toxicity by only 33% (**Figure 3C**). The same milk products decreased the toxic activity of Nhe reference strain NVH 0075-95, which was generally lower than the other two strains in this experiment, by approximately 40% (**Figure 3E**). These results suggest that haemolysin BL might be more affected by the presence of foodstuffs than the non-hemolytic enterotoxin.

Following these results, we were further interested which food components might be responsible for the reduction of the toxic effects. The tested substances are summarized in **Table 1**. As the baby follow-on formula had most impact on strain F837/76 (**Figure 3A**), this strain was used for investigating its ingredients. All tested substances are summarized in **Table 1**. Further components, fish oil and fruit powder, could not be tested. Substances with significant impact on cytotoxicity are shown in **Figure 3B**. A skim milk powder, vitamin C, vitamin B5 and vitamin A decreased the toxic activity, while enhanced toxicity titers were detected with skim milk powder III, biotin and vitamin B3. Milk proteins showed only minimal effects on the toxic activity of strains F837/76 and NVH 0075-95 (data not shown). On the other hand, α-casein, α-lactalbumin and especially lactoferrin decreased toxicity of the *nheABC* deletion strain (**Figure 3D**).

### Foodstuffs Partially Block Cell Binding, but Rather Interfere With the Interaction of the Single Toxin Components

**FIGURE 4:**
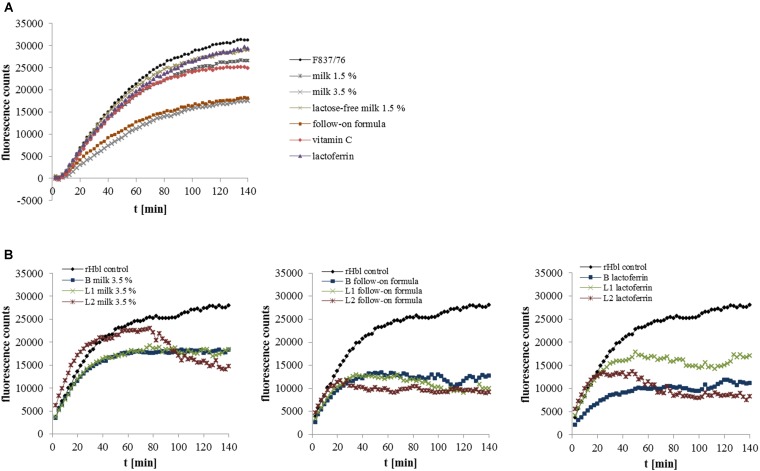
Pore forming activity of *B. cereus* enterotoxins determined in propidium iodide influx tests. **(A)** CaCo-2 cells were pre-incubated for 1 h with the different food and food components and washed three times in RPMI 1640 medium. Subsequently, supernatant of strain F837/76 was added in 1:40 dilution. **(B)** The recombinant Hbl components B, L1 and L2 were applied consecutively for 1 h each, either in RPMI 1640 medium or in the medium containing the food components in concentrations as shown in **Table 1**. After application of rHbl B and L1, CaCo-2 cells were washed three times. PI influx is shown as increase of fluorescence per time.

To get a first idea about the mechanism by which the different food components might impede enterotoxic activity, selected foods and toxin-containing *B. cereus* supernatants were applied to CaCo-2 cells consecutively. For consecutive application, PI influx tests are more suitable than WST-1 bioassays. Here, pore-forming activity of the enterotoxins can be observed in real time. Pore formation of F837/76 supernatant was rarely affected by pre-incubation with the different foodstuffs, only milk 3.5% and the baby follow-on formula resulted in delayed and less increase of fluorescence (**Figure 4A**), indicating that these two products might partially block the cell surface toward enterotoxin binding.

Simultaneously, food components with effect on toxic activity were mixed with the *B. cereus* supernatant in enzyme immunoassays detecting the single enterotoxin proteins. Certain food components altered the reciprocal titers obtained for NheA, Hbl L1 and Hbl B. These data are summarized in **Table 1**. The results indicate that some enterotoxin components might be blocked by the foodstuffs, and thus binding of the specific mAbs is altered. Hence, the interaction between the single enterotoxin components might also be influenced.

To investigate this in detail, the recombinant Hbl components B, L1 and L2 (Tausch et al., 2017) were applied in PI influx tests consecutively. One Hbl protein at a time was applied in RPMI 1640 medium mixed with selected foods. Milk 3.5%, the follow-on formula and lactoferrin showed the most impact on pore-forming activity (**Figure 4B**). For approximately 70 min, milk 3.5% had no influence on rHbl L2, only a late decline in fluorescence was observed. Activities of rHbl B and Ll were affected similarly. The follow-on formula hindered pore-formation most clearly, whatever Hbl component it was applied with. On the contrary, lactoferrin had the strongest impact on rHbl B activity.

## Discussion

In this study we demonstrated a significant impact of the consumed food on spore survival and cytotoxic activity of *B. cereus*. Although highly strain-specific, spores seemed to be protected from stomach medium and low pH by more complex milk products with high fat content (**Figures 1**, **2**). In a previous study is has already been shown that the number of spores incubated in gastric medium at low pH remained stable when half-skim milk or chicken were added (Clavel et al., 2004). Ceuppens and coworkers grew *B. cereus* in lasagne verde (Ceuppens et al., 2012b,c) before simulating the human upper gastrointestinal tract. As in our study, vegetative cells could not survive these conditions. Furthermore, the authors concluded that food can strain-specifically benefit spore survival (Ceuppens et al., 2012c). As a Gram-positive bacterium, *B. cereus* is highly sensitive toward bile salts, which can be relieved by different food components (Clavel et al., 2004; Ceuppens et al., 2012c).

The influence of food on the toxic activity of *B. cereus* is to date best investigated for kefir. It has been shown in cell toxicity assays, apoptosis and necrosis studies that kefir strain-specifically protects CaCo-2 cells from *B. cereus* culture supernatants (Medrano et al., 2008). Furthermore, cells “coated” with kefir were better preserved from detachment, F-actin disorganization and microvilli effacement. Kefir also modified the distribution of adhered *B. cereus*. The authors further demonstrated that the exopolysaccharide kefiran is able to interact with both eukaryotic cells and *B. cereus* (Medrano et al., 2009). In our study, milk and a baby follow-on formula strain-specifically reduced the toxic activity of *B. cereus* culture supernatants (**Figure 3**). Of the single milk components, lactoferrin showed the most inhibiting effects (**Figure 3D**). This iron-binding glycoprotein is an important component of the innate immune system, which has been reported to inhibit bacterial growth (Sato et al., 1999; Valenti et al., 2004; Ochoa and Cleary, 2009; Luna-Castro et al., 2014) as well as adhesion, invasion, motility, aggregation and biofilm formation (Valenti et al., 2004; Valenti and Antonini, 2005; Ochoa and Cleary, 2009; Luna-Castro et al., 2014). By binding LPS, it prevents inflammation, sepsis and gastroenteritis caused by enteric viruses, *Salmonella*, *Shigella*, or *E. coli* (Ochoa and Cleary, 2009; Latorre et al., 2010). It is also able to counteract cholera toxin by decreasing binding of the toxin to GM1-ganglioside on the target cell surface (Rivera et al., 2013). Cholera toxin belongs to the bacterial AB_5_ toxins (Merritt and Hol, 1995), which exhibit a mode of action different from the pore-forming *B. cereus* enterotoxins. Nevertheless, some evidence has been found that lactoferrin can also act as serine protease (Valenti et al., 2004), and might thus inhibit the highly protease-sensitive *B. cereus* enterotoxins. Of the single follow-on formula components, a skim milk powder and vitamins C, B5 and A showed inhibitory effects in our study (**Figure 3B**). Vitamin C is an antioxidant with important immunological function (Ströhle et al., 2011; Chambial et al., 2013; Sorice et al., 2014). A decreased risk of gastric diseases, also with reference to *Helicobacter pylori* infections, has been described (Tabak et al., 2003). Essential immunological functions are also attributed to vitamin A (Ströhle et al., 2011; Brown and Noelle, 2015; Sirisinha, 2015). Alpsoy and coworkers showed protective effects of vitamins A, C and E against aflatoxin B (Alpsoy et al., 2009a,b; Alpsoy and Yalvac, 2011). Furthermore, vitamin C decreases endothelial cell permeability (May and Qu, 2010, 2011), which might counteract the pore-forming *B. cereus* enterotoxins.

In this study the different foods only partially blocked the enterotoxins from binding to the target cell surface (**Figure 4A**). We rather found first evidence that the interaction between the single toxin components is disrupted. Under addition of different foods, detection of the single enterotoxin components in enzyme immunoassays was altered (see **Table 1**). The variability in binding of the specific mAbs is possibly due to blocking of the enterotoxin proteins by food components. Thus, it is, similarly, conceivable that such a “food-coated” enterotoxin protein is no longer able to interact with the other toxin components. This assumption was supported as foods also interfered with the sequential binding of recombinant Hbl components to the cell surface (**Figure 4B**).

*B. cereus* enterotoxins are unique among the pore-forming toxins, as they assemble from three different proteins. A specific binding order, i.e., NheC-B-A, is necessary for toxicity of the non-hemolytic enterotoxin (Sastalla et al., 2013). First, NheC and B, which already form complexes in solution, attach to the cell surface (Didier et al., 2012; Heilkenbrinker et al., 2013). In a second step, NheA binds, undergoes conformational changes and completes the pore (Didier et al., 2016). If NheA is blocked, which our data point to, the pore cannot be completely formed and its toxic effect is hindered. Similarly, the specific binding order B-L1-L2 is necessary for Hbl toxicity (Sastalla et al., 2013). Furthermore, the single Hbl components form complexes in solution (Tausch et al., 2017). Again, the pore might not be completely formed due to the blocking of single components. Altogether, the system of pore-formation by *B. cereus* enterotoxins is highly complex and thus, also highly vulnerable to disruptive factors such as protein- or cell-binding food components.

The present study is another step toward our overall goal to identify patterns for risk assessment of foods contaminated with enteropathogenic *B. cereus*. So far, the enteropathogenictype is found mainly in milk and meat products, vegetables or soups, while emetic strains appear mostly in rice, pastry or pasta (Kramer and Gilbert, 1989; Shinagawa, 1990). The different foods could be divided into risk categories, according to criteria such as likelihood of contamination (see above), promotion of bacterial survival and growth before consumption (Gilbert et al., 1974), facilitation of survival during stomach passage (Clavel et al., 2004; Ceuppens et al., 2012b,c and this study) as well as influence on cytotoxic activity (Medrano et al., 2008, 2009 and this study).

## Conclusion

Predicting the consumer’s risk of *B. cereus*-contaminated foods is extremely difficult, as it depends on various factors. First, on the particular *B. cereus* strain, which can be characterized in detail by now (Jessberger et al., 2015, 2017). Equally important is the type of food the bacteria are ingested with, as it influences not only the number of bacteria entering the small intestine, but also the cytotoxic effect of the enterotoxins toward epithelial cells. According to these findings, food products could be divided into different risk categories.

## Author Contributions

CDR was responsible for spore preparation as well as for survival and cytotoxicity assays. RD and EM were involved in experimental setup and writing of the manuscript. NJ also conducted cytotoxicity assays and wrote the manuscript.

## Conflict of Interest Statement

The authors declare that the research was conducted in the absence of any commercial or financial relationships that could be construed as a potential conflict of interest.
